# ST-T segment changes in prehospital emergency physicians in the field: a prospective observational trial

**DOI:** 10.1186/s13049-022-01033-1

**Published:** 2022-07-15

**Authors:** Mathias Maleczek, Karl Schebesta, Thomas Hamp, Achim Leo Burger, Thomas Pezawas, Mario Krammel, Bernhard Roessler

**Affiliations:** 1 Medical Simulation and Emergency Management Research Group, Department of Anaesthesia, Intensive Care Medicine and Pain Medicine, Medical University of Vienna, Waehringer Guertel 18-20, 1090 Vienna, Austria; 2 Academic Simulation Center of Vienna, Vienna, Austria; 3 Department of Medicine II, Division of Cardiology, Medical University of Vienna, Vienna, Austria; 4 Emergency Medical Service Vienna, Vienna, Austria; 5 PULS – Austrian Cardiac Arrest Awareness Association, Vienna, Austria

**Keywords:** ST-T segment, Stress, Emergency, Critical care, Prehospital

## Abstract

**Aims:**

Due to time-critical decision-making, physical strain and the uncontrolled environment, prehospital emergency management is frequently associated with high levels of stress in medical personnel. Stress has been known to cause ischemia like changes in electrocardiograms (ECGs), including arrhythmias and deviations in ST-T segments. There is a lack of knowledge regarding the occurrence of changes in ST-T segments in prehospital emergency physicians. We hypothesized that ST-T segment deviations occur in prehospital emergency physicians in the field.

**Methods:**

In this prospective observational trial, ST-T segments of emergency physicians were recorded using 12-lead Holter ECGs. The primary outcome parameter was defined as the incidence of ST-T segment changes greater than 0.1 mV in two corresponding leads for more than 30 s per 100 rescue missions. The secondary outcomes included T-wave inversions and ST-segment changes shorter than 30 s or smaller than 0.1 mV. Surrogate parameters of stress were measured using the NASA-Task Load Index and cognitive appraisal, and their correlation with ST-T segment changes were also assessed.

**Results:**

Data from 20 physicians in 36 shifts (18 days, 18 nights) including 208 missions were analysed. Seventy percent of previously healthy emergency physicians had at least one ECG abnormality; the mean duration of these changes was 30 s. Significantly more missions with ECG changes were found during night than day shifts (39 vs. 17%, p < 0.001). Forty-nine ECG changes occurred between missions. No ST-T segment changes > 30 s and > 0.1 mV were found. Two ST-T segment changes < 30 s or < 0.1 mV (each during missions) and 122 episodes of T-wave inversions (74 during missions) were identified. ECG changes were found to be associated with alarms when asleep and NASA task load index.

**Conclusion:**

ECG changes are frequent and occur in most healthy prehospital emergency physicians. Even when occurring for less than 30 s, such changes are important signs for high levels of stress. The long-term impact of these changes needs further investigation.

*Trial registration* The trial was registered at ClinicalTrials.gov (NCT04003883) on 1.7.2019: https://clinicaltrials.gov/ct2/show/NCT04003883?term=emergency+physician&rank=2

## Background

Emergency physicians are exposed to high levels of physical and psychological stress [1–3]. This is due to high-stakes medical decision-making, time constraints and working out of hours. Furthermore, physically challenging access to the patients and constantly changing teams can contribute to stress [4]. Electrocardiographic (ECG) changes can be a sign of stress with a broad spectrum of effects, including elevated risk for cardiac events, burnout and fatique [5, 6]. It has been shown that physical and mental stress leads to changes in the ST-T segment. Changes in ST-T segment generally are seen as sign of coronary ischaemia [7, 8]. Among airline pilots with previously known significant coronary disease, 25% developed ST segment changes during aviation mental stress tests [9, 10]. Among marathon runners, 7.5% of participants without known cardiac disease showed ST-T segment changes, and 8% of participants in a ski marathon showed ST trace depressions of variable duration. Although an increase in troponin was observed after these events in marathon participants, the five-year follow-up did not reveal a higher rate of cardiac events such as myocardial infarction, arrhythmias, or death [11, 12]. ST-T segment changes have not been reported in medical personnel until now. Prehospital emergency care is a high-stakes domain with exposure to increased levels of stress. In this trial, we hypothesized that ST-T segment changes occur in healthy emergency physicians during prehospital emergency care.

## Methods

### Study design

A prospective single-blinded observational trial was conducted. It was registered at ClinicalTrials.gov (NCT04003883). Outcomes including the definition of corresponding leads were defined following the European Society of Cardiology’s (ESC) guidelines on acute myocardial infarction [7, 8]. The primary outcome of this trial was the incidence of ST-T segment change > 0.1 mV for more than 30 s in two corresponding leads per 100 missions. As secondary outcomes, potential indicators of ischaemia were used: Incidences of (a) changes in ST-T segments < 0.1 mV or < 30 s) per 100 missions, (b) new onset T-wave inversions for more than 30 s per 100 missions, and c) T-wave inversions for less than 30 s per 100 missions.

Furthermore, we assessed correlation of influencing parameters on ECG changes: (1) The different phases of missions to the ECG changes listed above, (2) correlation of the ten most stressful alarm codes to ECG changes and (3) special events logged (Intubation, pediatric emergency,…) to ECG changes.

To assess the psychological stress during mission the National Aeronautics and Space Administration Task Load Index (NASA-TLX) and Cognitive appraisal was used [16–18]. The correlation of the 10 most stressful and lesser stressful mission codes to surrogate parameters of stress measured using NASA-TLX and to cognitive appraisal was assessed.

### Population

The included emergency physicians were anaesthesiologists, emergency medicine consultants and senior anaesthesia or emergency medical residents with prehospital emergency medicine credentials and no previously known underlying cardiovascular diseases.

The exclusion criteria were as follows: known pregnancy, pre-existing cardiac diseases (valvular heart disease > I°), any form of cardiomyopathy or channelopathy diagnosable with ECG, echo or ergometry, history of coronary artery disease, history of myocarditis, known high degree (> 1% of all beats within 24 h) premature atrial or ventricular beats or atrial fibrillation or conduction disturbance, any antiarrhythmic therapy, any implanted cardiac device and manifest hyperthyroidism.

Written informed consent was obtained from every participant prior to data collection.

To ensure that no pre-existing cardiologic pathologies were present, every participant was tested, including a medical history, a 12-lead resting ECG, a transthoracic echocardiography and a 24-h ECG during a day off as well as an ergometry. Participants with abnormal test results indicating relevant cardiac pathologies were excluded from the trial and referred to the cardiology department.

### Data obtainment

The advanced life support unit at the Medical University of Vienna is manned by an emergency physician from mainly the anaesthesia department or the emergency medicine department and a paramedic from the Medical Emergency Service Vienna. Shifts lasted approximately 8–16 h: Dayshifts lasted between 8 and 12 h depending on the day of the week and nightshifts lasted 12–16 h. The emergency physician was alerted via pager/mobile phone when at the station and via an electronic alert at the vehicle’s computer terminal while in the response car. Both alerts sound a loud signalling noise. On mobile phone and computer terminal the missions information are displayed including place of emergency and basic information about the call.

A 12-lead Holter ECG (FD12 + , Schiller AG, Switzerland) with Ambu BlueSensor VL ECG electrodes (Ambu A/S, Ballerup, Denmark) was used. To ensure signal quality, theHolter ECG was tested during standardized ergometry to validate the measurements using a GE e-bike comfort ergometer (GE Medical Systems, e-bike comfort Series 1, MI, USA) by a senior cardiologist (TP). No deviations occurred.

ECGs were recorded for one day and one night shift for each participant. After arrival at the ambulance station, the participants were asked to take 10 min to relax, after which the ECG was attached. Electrodes were placed in a standard 12-lead formation. During the shift, participants were asked to write a log about the missions. This log contained information about the diagnosis of the patients treated, patient age (< or ≥ 18 years), special events, and procedures (intubation, i.v.-medication, cardiopulmonary resuscitation, and other invasive procedures). Additionally, chest pain experienced by the participant was recorded. Participants were asked to mark if alarms were received during sleep or while awake. All participants were instructed to use the pager alarm system, as it is local standard practice for alerts during the night to create standardized conditions.

### Surrogate parameter of stress

Surrogate parameters of stress were obtained by using cognitive appraisal and TLX. Cognitive appraisal was measured en route to the patient and after handover of the patient using the method described by Tomaka et al. by dividing the expected (primary) appraisal and the real (secondary) appraisal using a 10-point Likert-like scale, the cognitive appraisal index was calculated. An index < 1 indicates that resources did not meet demands, and the task is appraised as a “threat”, while an index > 1, where resources were greater than demands, indicates a “challenge” [17, 18].

The NASA-TLX is widely used in health care and was developed to assess the workload of a task across six subscales: Mental Demand, Physical Demand, Temporal Demand, Performance, Effort and Frustration [19–23]. The NASA-TLX was measured after the mission to assess the participant’s individual workload.

### Data analysis

After shifts, the ECG and the participant’s logs were collected and saved for analysis. ECG analysis was conducted by MM supervised by a senior cardiologist (TP).

Investigators analysing the ECGs were blinded to the participant’s names and details of missions, including the logs. ECGs were analysed after recording using the software supplied by the manufacturer (medilog DarwinV2 2.*—Schiller AG, Switzerland, 2017) with the aim of identifying ST-T segment changes, T-wave inversions and other ECG abnormalities.

Missions were divided into four phases: alarm (two minutes before the alarm until confirmation of alarm), en route (while en route to patient), patient care (arrival at the patient until departure from scene), and transport to hospital (if done).

The ten most stressful alarm codes were identified preliminarily via a modified Delphi process [24, 25].

### Statistical analyses

According to previous data and unpublished local observations during a pilot phase of the project, the incidence of significant ECG changes defined ST-T segment changes > 0.1 mV and > 30 s as was expected to occur in 10% of all prehospital emergency response missions in physicians [11, 12]. As the workload during shifts is heterogeneous, we used a convenience sample of 25 physicians in each shift (day, night), resulting in 50 shifts (300 expected missions, range: 150–600).

For all primary and secondary analyses, prehospital emergency response missions were considered as the unit of observation all measurements were standardized to. Descriptive statistics such as the mean and standard deviation were computed for all metric variables. Absolute and relative frequencies were calculated for categorical variables. Descriptive statistics were computed for the overall data and for each grouping variables.

A two-sided Student’s t-test and chi-square test were used as appropriate to assess relations between primary and secondary as well as within secondary outcome parameters. Secondary outcome parameters were used to generate hypotheses. Therefore, no correction for multiple testing was performed, and p values < 0.05 were considered statistically significant. All analyses were performed using Python 3.8, mainly the pandas and numpy packages [26, 27].

Ethical approval of the Medical University of Vienna’s Institutional Review Board (EK 1648/19), the Workers’ Council and Data Protection Commission was given prior to inclusion of the first proband.

## Results

**Fig. 1:**
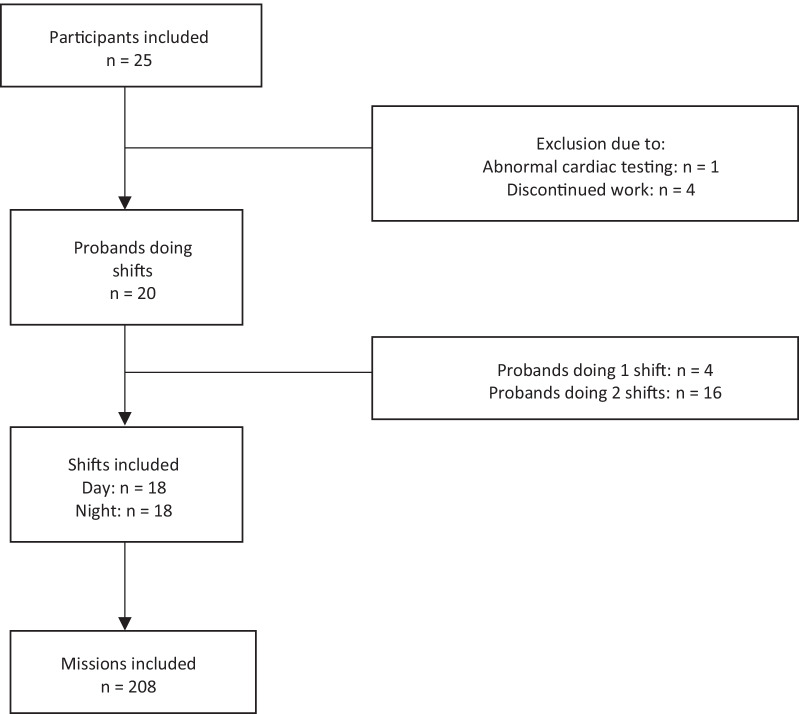
Prisma flowchart. A Prisma flowchart of the participants including dropouts is shown

The study population consisted of 25 emergency physicians. After the cardiac tests, one physician had to be excluded due to a pre-existing ventricular septal defect resulting in ECG abnormalities. All others had a normal resting ECG, ergometry, echocardiography, 24 h Holter ECG during off-duty time. During the study period, four more physicians had to be excluded because they stopped doing their shifts in preclinical emergency medicine due to paternity leave, illness or changing place of work, resulting in 20 emergency physicians included in the study. (Fig. 1) Except for 4 physicians who only did one shift, all others did one night and one day shift, resulting in 36 recorded (18 day and 18 night) shifts with a total of 208 missions between 2019-11-15 and 2021-03-27. Details can be found in Table 1.

**Table 1:** Demographic details of the included emergency physicians and their missions

Emergency physicians (n)	20
Age mean (SD)	39.1 (4.1)
Male n (%)	12 (60%)
Shifts (n)	36
Missions n (mean^*^)	208 (5.8)
Missions during nightshifts n (mean^#^)	80 (4.4)
Missions during dayshifts n (mean^#^)	128 (7.1)

*  Mean missions per shift, ^#^mean number of missions

### ECG findings

Seventy percent of emergency physicians had at least one ECG change during their shifts. Significantly more shifts with at least one ECG change were observed during night shifts (38.8%) than during day shifts (17.2%) (chi2: p < 0.001).

In total, 124 ECG changes were found. Of those, 75 ECG changes occurred during missions and 49 between missions. During 15 shifts, more than one ECG change was observed. The mean duration of those changes was 30 s (min/max: 3 s/497 s). The primary outcome of ST segment changes in > 2 leads with > 30 s duration could not be found. One ST-T segment change of < 30 s and > 0.1 mV and one > 30 s but < 0.1 mV were observed (0.5/100 missions each). Most of the changes (122, 98.4%, 59/100 missions) were temporary T-wave inversions. Twenty-seven (13/100 missions) of those changes met the secondary outcome criteria of > 30 s duration in 95 (48/100 missions) T-wave inversions was ≤ 30 s. More details can be found in Table 2.

**Table 2:** Details of ECG changes and their distribution between shifts and the 208 missions

ECG finding	Total number n	Number during dayshift n (%)	Number during Nightshifts n (%)	ECG changes during missions n (n/100 missions)
ST change > 30 s, > 0.1 mV	0	0	0	0
ST change < 30 s or < 0.1 mV	2	2 (100%)	0	1 (0.5)
T-wave invers. > 30 s	27	7 (26%)	20 (74%)	13 (6.25)
T-wave invers. ≤ 30 s	95	36 (37.9%)	59 (62.1%)	61 (29.3)
Total	124	45 (31.3%)	79 (68.8%)	75 (36.1)

**Fig. 2:**
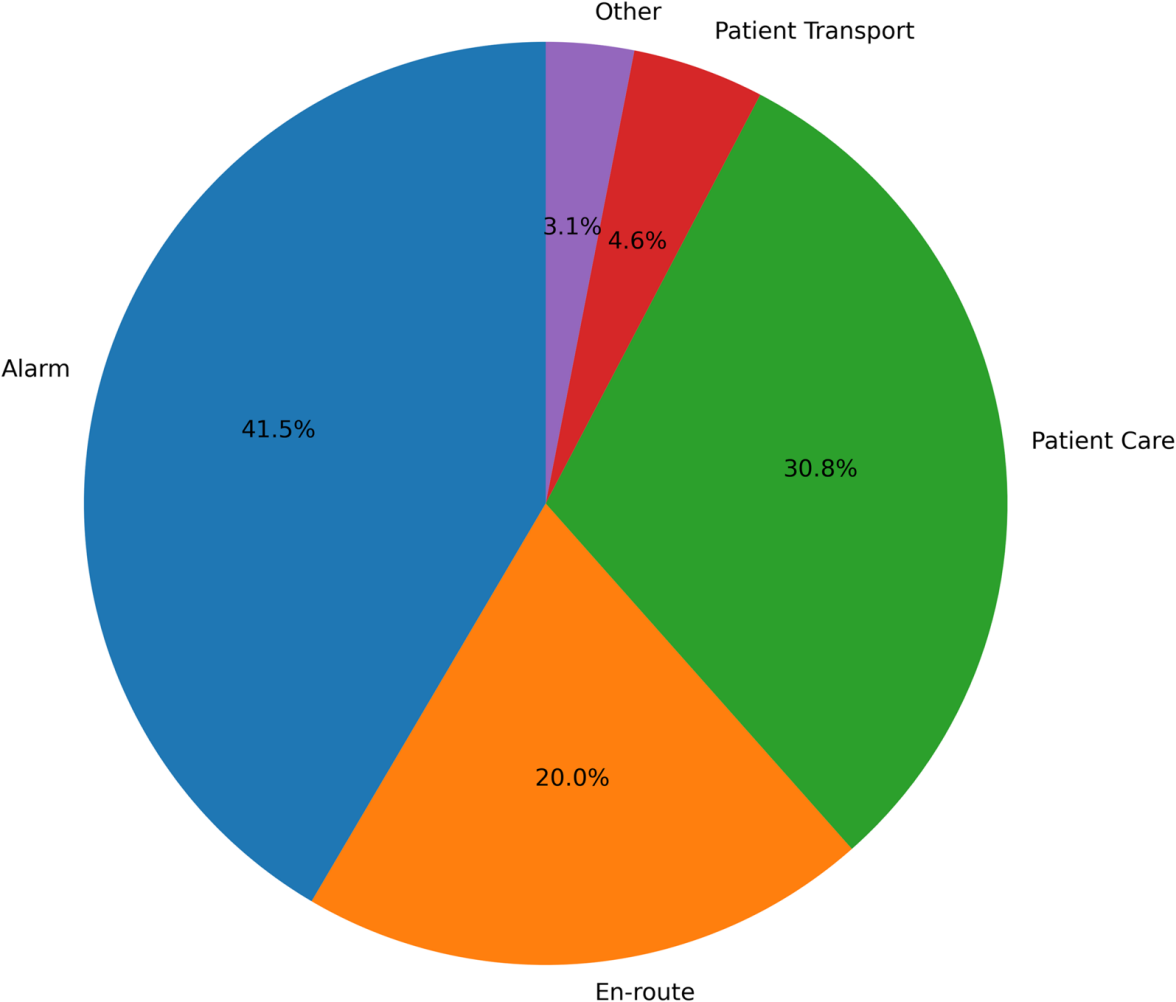
Distribution of ECG changes. The distribution of ECG changes between phases of a mission is shown. En-route is driving to the patient, patient care is the time between arrival on scene and start of patient transport

ECG changes were not distributed equally between the four phases of a mission: alarm, drive to patient (en-route), patient care, and transport to hospital (p < 0.001). The majority of ECG findings occurred during the alarm phase (41.5%), followed by the patient care (30.8%), en route phases (20.0%) and patient transport (4.6%). A total of 3.1% of changes occurred within 5 min after mission and therefore were not assigned to one of the predefined phases such as patient care. (Fig. 2).

In the 24-h Holter ECGs, one ECG change (T wave inversion) was found. In the log, the participant noted that he was woken by the washing machine’s alarm in the middle of the night.

Overall, the signal quality with the used Holter was excellent with only one episode of missing registration occurring.

### Surrogate parameters of stress

Data on cognitive load were available in 167 missions (80%), and NASA-TLX was available in 192 missions (92%).

**Fig. 3:**
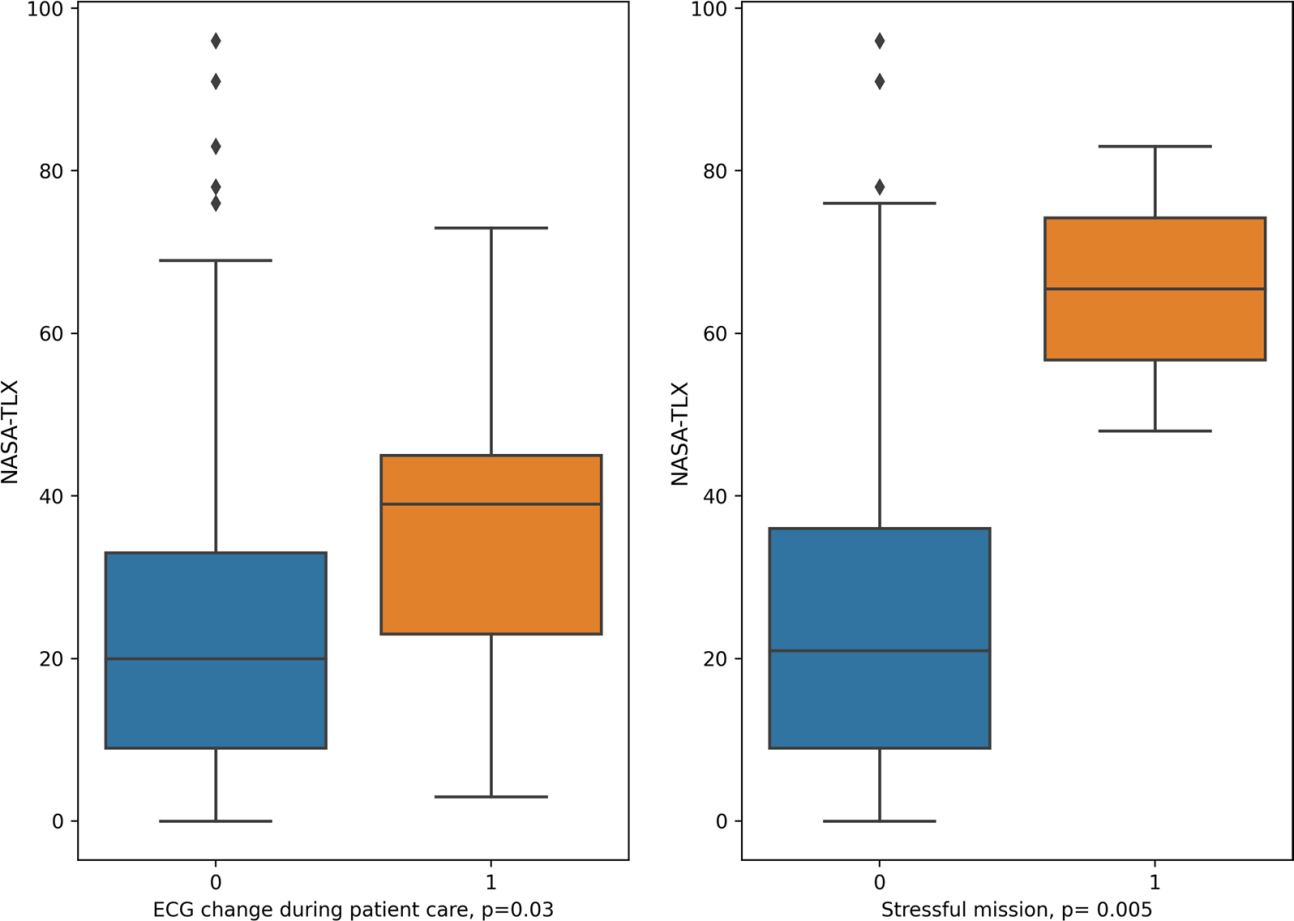
Differences in NASA-TLX. The boxplot on the left shows NASA-TLX of a mission by any ECG change occurring during patient care. The boxplot on the right shows NASA-TLX classified by most stressful alarm codes as defined by a Delphi process

The mean cognitive load was 0.6 (SD: 0.67), indicating that the missions were mainly perceived as challeng, not as threat. The mean NASA-TLX score was low, at 25.6 (SD: 20.5). No significant correlation between missions with ECG changes and cognitive load or TLX were found (CL: *t*-test, p = 0.4, TLX: *t*-test, p = 0.4) when including all missions. When considering only the emergency physicians who had ECG changes during patient care, scores on the NASA-TLX were significantly higher when ECG abnormalities were present (t-test, p = 0.03, Fig. 3) as well as in missions classified as stressful in the preliminary Delphi process (t-test, p < 0.01, Fig. 3).

The ten alarm codes perceived to be most stressful by emergency physicians are presented in Table 3.

**Table 3:** The table shows the ten mission codes classified most stressfully by the Delphi process

Code	Text
24D01	Pregnancy-breech or cord
17D01	Fall-Extreme fall (≥ 10 m)
24D06	Pregnancy-baby born (complications with baby)
11E01	Suffocation-complete obstruction/ineffective breathing
07E01	Burn-person on fire
27D01	Penetrating injury. Cardiac arrest
04D04	Assault-chest or neck injury (with difficulty breathing)
02E01	Allergy-ineffective breathing
22D01	Inaccessible incident-entrapment
11D01	Suffocation-abnormal breathing (partial obstruction)

As mission alarm codes, the advanced medical dispatch system is used

Of the 208 studied missions, only two were stressful codes. During those two missions, no ECG changes occurred.

The events marked by the physicians (paediatric emergency, iv medication, intubation and polytrauma) had no significant association with ECG changes (chi2 p > 0.05). When the alarm occurred during a sleep phase significantly more participants had ECG changes (chi2, p = 0.001).

## Discussion

Prehospital emergency medicine is both physically and psychologically challenging, leading to relevant ECG changes. The incidence of ST-T segment changes remains unclear; therefore, the aim of this trial was to show the incidence of ST-T deviations and other ECG changes, such as T wave inversions, in emergency physicians.

By recording ECGs during a shift to assess emergency physicians, we aimed to close this knowledge gap. In contrast to the primary hypothesis, we found no significant ST-T deviation that fulfilled primary outcome criteria as defined by the ESC. Nevertheless, minor ST-T deviations and a considerably high number of T-wave inversions could be detected frequently especially during missions at night. A high number of T-wave inversions was seen between missions. However, these changes did not correlate with predefined stressful codes.

To our knowledge, this is the first trial investigating ST-T deviations in healthy prehospital emergency physicians while on shift. In contrast to the previously published trial by Doorey focusing on pilots with known coronary artery disease showing signs of ischemia during stress, this trial concentrated on participating physicians without pathological cardiac history. Even in this population, ST-T deviations and T-wave inversions were frequent.

It is commonly accepted, that ST-T segment deviations are typically caused by ischemia [10]. While the follow-up of the marathon trial observing ST deviations during running showed no increased incidence of cardiac events after one yearsilent ST-T changes in exercise testing were linked to an increased risk of cardiac death [11, 28, 29].

Another factor known to cause changes in the ST-T segment is stress [9–12, 30, 31]. However, the effect of stress-induced ST-T segment changes is not fully understood.

It seems likely that the reported ECG changes are partly attributed to stress. This is supported by the fact that most changes were seen during the alarm and patient care phases, when there was a combination of psychological and physical stress. Whether this stress results from the stress induced by the loud noise of the pager, sudden awakening, rapid change into an upright position or psychological stress induced by patient care remains unclear. In a tilt-table test, it was shown that rapid change in position can cause ST-T depression and T-wave inversion even in patients with no known cardiac disease [31]. As the volunteers/participants had these changes not only when sitting up, not all changes can be explained by this.

A rather large portion of the ECG changes occurred while treating a patient. During this time interval, many different stressors occurring during alarm are not present. Therefore, it seems likely that the ECG changes reflect stress.

A further possible explanation for stress induced ST-T segment changes is an autonomous conflict between sympathic and parasympathic responses, which can be shown especially during abrupt wake up from deep sleep. Shattock MJ. et al studied this by immersing participants in cold water [32]. ECG changes in our trial that where observed when the alarm occurred during sleep might be attributed to such an effect.

Certain limitations of this study must be acknowledged. This trial was a single-blinded, single-centre observational study. ECGs were analysed by hand with the support of software. This absence of a consistent four eye principle may have led to some ECG changes being missed or overinterpreted. To reduce the possibility of bias, a senior cardiologist revaluated borderline ECG changes and reviewed a sample of the ECG in a routine way. Another possible limitation is the participant’s coronary risk. We tried to minimize this bias by performing extensive testing (ECG, 24-h ECG, echocardiography, blood samples, ergometry). Indeed, the authors had to exclude one participant due to abnormalities in the 24 h baseline ECG.

Due to the setting in preclinical emergency medicine, the conditions were not standardized. Stress is a very individual parameter representing an important limitation. By using the NASA-TLX and cognitive appraisal, we aimed to quantify these different stress levels. Furthermore, this trial examined a single observation of each participant’s cardiovascular response to stress. As more invasive measurements like serial troponins were not possible in the trials setting, this data were not collected and needs to be assess in future trials. Therefore our results are hypothesis generating by nature.

Emergency physicians at our centre are very highly trained and are able to work from a point of health and of similar socioeconomic state – this makes the population a very homogenous group, which is another limitation of this study.

Due to COVID, the study had to be paused in the beginning of the COVID pandemic due to roster changes and concerns of the hygiene authority of handing over the 12 Lead ECG from physician to physician (16/3/2020 – 1/6/2020). This and the duty roster of the participants led to a rather long study period of 17 month in total. During that time the length of shifts changed from 12 h + 12 h (day/night) to 8 h + 16 h (day/night).

As in the general Austrian emergency physician’s population 60% of the study’s population was male. A gender difference is known regarding risk factors, symptoms and diagnosis of ischemic heart disease [13]. This also includes differences in ECG changes, especially prevalence and type of signs of ischemia [14, 15].

The origin of the ECG changes cannot be definitively identified in this trial; therefore, further trials will be necessary to determine the origin of these changes. Important questions remain regarding the long-term impact of these ECG changes. To quantify whether these changes in the ST-T segment can help identify individuals at risk of cardiac adverse events, a long-term analysis is needed. An analysis including troponin to detect ischemic damage to heart muscle will be needed to solve the definite impact of these ECG changes on a cellular level.

Using our data, it will be possible to conduct interventional trials to understand the reasons for and regarding methods to reduce occupational stress. Stress levels are obviously very high in preclinical emergency medicine. Our trial provides data that ST-T segment changes – especially T-wave inversions – are common in medical professionals working in high-stakes environments.

## Conclusion

In our group of healthy emergency physicians, ECG abnormalities with a possible ischemic reason were frequently seen – mostly T-wave inversions. At least one ECG abnormality was found in 70% of the included emergency physicians. Alarm when sleeping was significantly associated with ECG changes. There was a significant association between the NASA task load index and changes in the ECG, showing the impact of stress on such changes.

## Data Availability

The datasets generated and/or analysed during th current study are not publicly available due to data safety restriction but are available from the corresponding author on reasonable request.

## Abbreviations

ECG: Electrocardiogram
NASA-TLX: National aeronautics and space administration task load index
proBNP: Pro brain natriuretic peptide
TSH: Thyroid stimulating hormone
